# Crystal structure of ethyl 2-[2-((1*E*)-{(1*E*)-2-[2-(2-eth­oxy-2-oxoeth­oxy)benzyl­idene]hydrazin-1-yl­idene}meth­yl)phen­oxy]acetate

**DOI:** 10.1107/S2056989014025584

**Published:** 2015-01-01

**Authors:** Joel T. Mague, Shaaban K. Mohamed, Mehmet Akkurt, Eman A. Ahmed, Omran A. Omran

**Affiliations:** a Department of Chemistry, Tulane University, New Orleans, LA 70118, USA; b Chemistry and Environmental Division, Manchester Metropolitan University, Manchester M1 5GD, England; c Chemistry Department, Faculty of Science, Minia University, 61519 El-Minia, Egypt; d Department of Physics, Faculty of Sciences, Erciyes University, 38039 Kayseri, Turkey; e Chemistry Department, Faculty of Science, Sohag University, 82524 Sohag, Egypt

**Keywords:** crystal structure, azomethenes, bis-phen­oxy carboxyl­ate

## Abstract

The complete mol­ecule of the title compound, C_22_H_24_N_2_O_6_, is generated by crystallographic inversion symmetry and is approximately planar (r.m.s. deviation of the non-H atoms = 0.134 Å). The packing consists of inter-digitated sheets inclined at 25.9 (4)° to one another and linked by short C—H⋯O hydrogen bonds.

## Related literature   

For background to the properties and applications of imines see: Sun *et al.* (2001 ▸); Boghaei & Mohebi (2002 ▸); Liu *et al.* (2006 ▸); Britovsek *et al.* (2001 ▸); Budakoti *et al.* (2006 ▸).
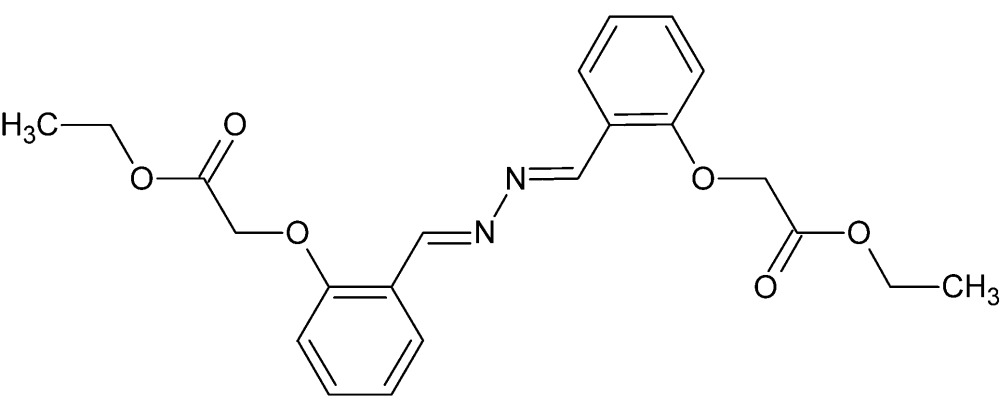


## Experimental   

### Crystal data  

C_22_H_24_N_2_O_6_
*M* *_r_* = 412.43
Monoclinic, 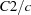

*a* = 18.2073 (5) Å
*b* = 11.7758 (3) Å
*c* = 9.9950 (3) Å
β = 93.226 (1)°
*V* = 2139.59 (10) Å^3^
*Z* = 4
Cu *K*α radiation
μ = 0.78 mm^−1^
*T* = 150 K
0.16 × 0.15 × 0.07 mm

### Data collection  

Bruker D8 VENTURE PHOTON 100 CMOS diffractometer
Absorption correction: multi-scan (*SADABS*; Bruker, 2014 ▸) *T*_min_ = 0.88, *T*_max_ = 0.94
12339 measured reflections
2124 independent reflections
1801 reflections with *I* > 2σ(*I*)
*R*_int_ = 0.032

### Refinement  

*R*[*F* ^2^ > 2σ(*F* ^2^)] = 0.035
*wR*(*F* ^2^) = 0.094
*S* = 1.06
2124 reflections
137 parameters
H-atom parameters constrained
Δρ_max_ = 0.19 e Å^−3^
Δρ_min_ = −0.21 e Å^−3^

### 

Data collection: *APEX2* (Bruker, 2014 ▸); cell refinement: *SAINT* (Bruker, 2014 ▸); data reduction: *SAINT*; program(s) used to solve structure: *SHELXT* (Bruker, 2014 ▸); program(s) used to refine structure: *SHELXL2014* (Sheldrick, 2008 ▸); molecular graphics: *DIAMOND* (Brandenburg & Putz, 2012 ▸); software used to prepare material for publication: *SHELXTL* (Bruker, 2014 ▸).

## Supplementary Material

Crystal structure: contains datablock(s) global, I. DOI: 10.1107/S2056989014025584/hb7321sup1.cif

Structure factors: contains datablock(s) I. DOI: 10.1107/S2056989014025584/hb7321Isup2.hkl

Supporting information file. DOI: 10.1107/S2056989014025584/hb7321Isup3.cml

**Fig. 1.**
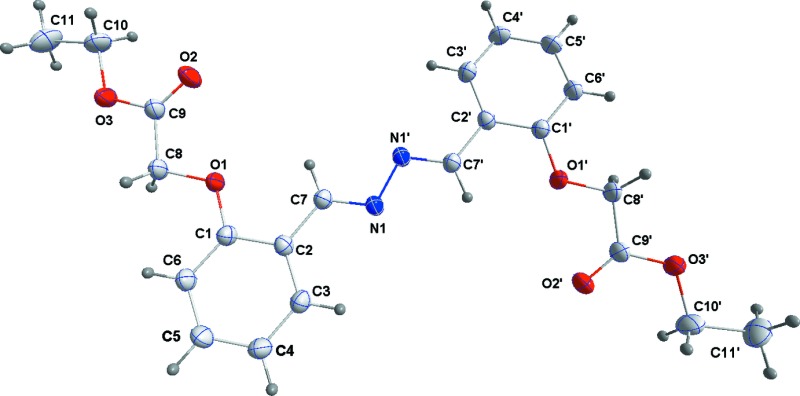
The title compound showing 50% probability ellipsoids. Primed atoms are related to their unprimed counterparts by the crystallographic center.

**Fig. 2.**
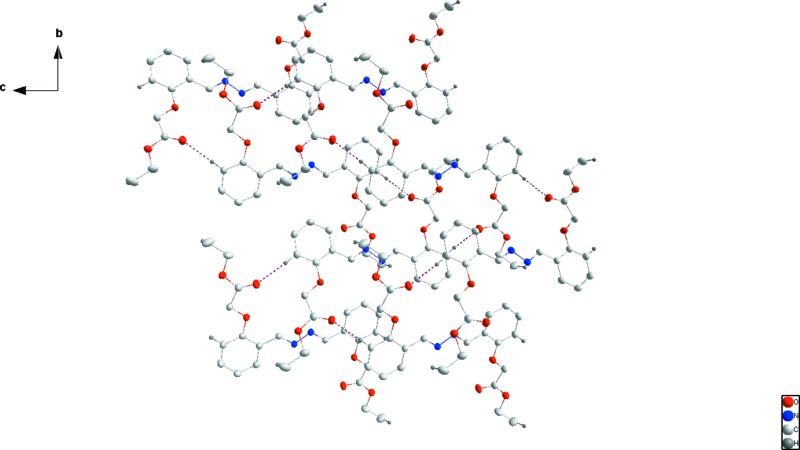
Packing viewed down the a axis with C—H···O interactions shown by dotted lines.

. DOI: 10.1107/S2056989014025584/hb7321fig1.tif
The title compound showing 50% probability ellipsoids. Primed atoms are related to their unprimed counterparts by the crystallographic center.

a . DOI: 10.1107/S2056989014025584/hb7321fig2.tif
Packing viewed down the *a* axis with C—H⋯O inter­actions shown by dotted lines.

**Fig. 3.**
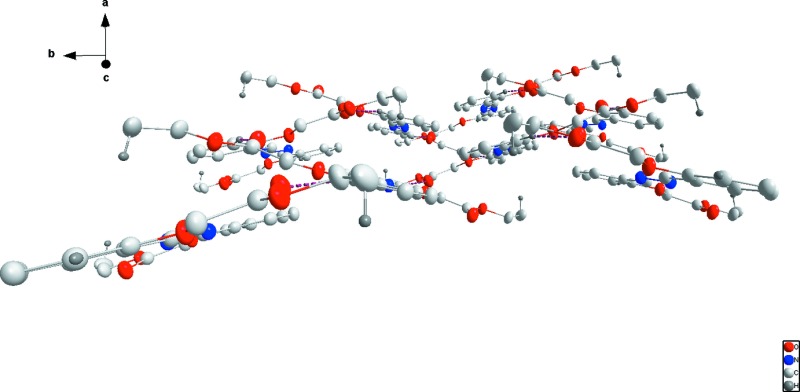
Elevation view of the interpentrating layer packing.

. DOI: 10.1107/S2056989014025584/hb7321fig3.tif
Elevation view of the inter­pentrating layer packing.

CCDC reference: 1035485

Additional supporting information:  crystallographic information; 3D view; checkCIF report

## Figures and Tables

**Table 1:** Hydrogen-bond geometry (, )

*D*H*A*	*D*H	H*A*	*D* *A*	*D*H*A*
C6H6O2^i^	0.95	2.34	3.2802(14)	168

Symmetry code: (i) 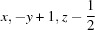.

## supplementary crystallographic information

### S1. Comment

Imines are used as catalysts, in medicine as antibiotics and anti-inflammatory agents and in industry as anticorrosion agents (Sun *et al.*, 2001; Boghaei & Mohebi, 2002; Liu *et al.*, 2006; Britovsek *et al.*, 2001; Budakoti *et al.*, 2006). Based on these finding we report here the synthesis and crystal structure of the title compound.

The title molecule has crystallographically imposed centrosymmetry with an "extended" conformation in which the central portion is almost planar. Intermolecular C6—H6···O2 hydrogen bonds (Table 1) assemble the molecules into interpenetrating sheets which are inclined to (100) by 24.0 and 24.3° and to one another by 25.9° (Figs. 2 and 3).

### S2. Experimental

A mixture of 1 mmol (326 mg) of ethyl (2-{(*Z*)-[(2*E*)-(2-hydroxybenzylidene)hydrazono]methyl}phenoxy)acetate and 1 mmol (167 mg) of ethyl bromoacetate in 30 ml of ethanol was heated under reflux for 24 h. The resulting solid product was filtered off, dried and recrystallized from dichlomethane solution to furnish pale yellow blocks in 90% yield (m.p. 383 K).

### S3. Refinement

H-atoms were placed in calculated positions (C—H = 0.95 - 0.98 Å) and included as riding contributions with isotropic displacement parameters 1.2 - 1.5 times those of the attached carbon atoms.

### Crystal data

**Table:**

C_22_H_24_N_2_O_6_	*F*(000) = 872
*M**_r_* = 412.43	*D*_x_ = 1.280 Mg m^−^^3^
Monoclinic, *C*2/*c*	Cu *K*α radiation, λ = 1.54178 Å
*a* = 18.2073 (5) Å	Cell parameters from 7734 reflections
*b* = 11.7758 (3) Å	θ = 4.5–72.3°
*c* = 9.9950 (3) Å	µ = 0.78 mm^−^^1^
β = 93.226 (1)°	*T* = 150 K
*V* = 2139.59 (10) Å^3^	Block, pale yellow
*Z* = 4	0.16 × 0.15 × 0.07 mm

### Data collection

**Table:**

Bruker D8 VENTURE PHOTON 100 CMOS diffractometer	2124 independent reflections
Radiation source: INCOATEC IµS micro-focus source	1801 reflections with *I* > 2σ(*I*)
Mirror monochromator	*R*_int_ = 0.032
Detector resolution: 10.4167 pixels mm^-1^	θ_max_ = 72.3°, θ_min_ = 4.5°
ω scans	*h* = −22→22
Absorption correction: multi-scan (*SADABS*; Bruker, 2014)	*k* = −13→14
*T*_min_ = 0.88, *T*_max_ = 0.94	*l* = −12→12
12339 measured reflections	

### Refinement

**Table:**

Refinement on *F*^2^	Primary atom site location: structure-invariant direct methods
Least-squares matrix: full	Secondary atom site location: difference Fourier map
*R*[*F*^2^ > 2σ(*F*^2^)] = 0.035	Hydrogen site location: inferred from neighbouring sites
*wR*(*F*^2^) = 0.094	H-atom parameters constrained
*S* = 1.06	*w* = 1/[σ^2^(*F*_o_^2^) + (0.0498*P*)^2^ + 0.7328*P*] where *P* = (*F*_o_^2^ + 2*F*_c_^2^)/3
2124 reflections	(Δ/σ)_max_ < 0.001
137 parameters	Δρ_max_ = 0.19 e Å^−^^3^
0 restraints	Δρ_min_ = −0.21 e Å^−^^3^

### Special details

**Table:**

Geometry. All e.s.d.'s (except the e.s.d. in the dihedral angle between two l.s. planes) are estimated using the full covariance matrix. The cell e.s.d.'s are taken into account individually in the estimation of e.s.d.'s in distances, angles and torsion angles; correlations between e.s.d.'s in cell parameters are only used when they are defined by crystal symmetry. An approximate (isotropic) treatment of cell e.s.d.'s is used for estimating e.s.d.'s involving l.s. planes.
Refinement. Refinement of *F*^2^ against ALL reflections. The weighted *R*-factor *wR* and goodness of fit *S* are based on *F*^2^, conventional *R*-factors *R* are based on *F*, with *F* set to zero for negative *F*^2^. The threshold expression of *F*^2^ > σ(*F*^2^) is used only for calculating *R*-factors(gt) *etc*. and is not relevant to the choice of reflections for refinement. *R*-factors based on *F*^2^ are statistically about twice as large as those based on *F*, and *R*- factors based on ALL data will be even larger. H-atoms were placed in calculated positions (C—H = 0.95 - 0.98 Å) and included as riding contributions with isotropic displacement parameters 1.2 - 1.5 times those of the attached carbon atoms.

### Fractional atomic coordinates and isotropic or equivalent isotropic displacement parameters (Å^2^)

**Table:**

	*x*	*y*	*z*	*U*_iso_*/*U*_eq_	
O1	0.65269 (5)	0.39734 (7)	0.64113 (8)	0.0303 (2)	
O2	0.60379 (5)	0.59761 (8)	0.72472 (8)	0.0394 (2)	
O3	0.57361 (5)	0.65089 (7)	0.51290 (8)	0.0310 (2)	
N1	0.74455 (5)	0.21539 (8)	0.94262 (9)	0.0274 (2)	
C1	0.66504 (6)	0.28591 (9)	0.60919 (11)	0.0247 (2)	
C2	0.69652 (6)	0.21893 (9)	0.71363 (10)	0.0240 (2)	
C3	0.70953 (6)	0.10397 (10)	0.68869 (12)	0.0283 (3)	
H3	0.7311	0.0577	0.7581	0.034*	
C4	0.69152 (7)	0.05673 (10)	0.56450 (12)	0.0314 (3)	
H4	0.6996	−0.0218	0.5492	0.038*	
C5	0.66146 (7)	0.12522 (11)	0.46220 (12)	0.0308 (3)	
H5	0.6497	0.0930	0.3765	0.037*	
C6	0.64838 (6)	0.23969 (10)	0.48302 (11)	0.0277 (3)	
H6	0.6283	0.2859	0.4121	0.033*	
C7	0.71462 (6)	0.27139 (9)	0.84382 (11)	0.0253 (2)	
H7	0.7039	0.3496	0.8557	0.030*	
C8	0.61552 (7)	0.46587 (10)	0.54257 (11)	0.0284 (3)	
H8A	0.5696	0.4282	0.5085	0.034*	
H8B	0.6470	0.4784	0.4664	0.034*	
C9	0.59814 (6)	0.57753 (10)	0.60731 (11)	0.0274 (3)	
C10	0.55682 (7)	0.76451 (11)	0.55916 (13)	0.0378 (3)	
H10A	0.6004	0.7979	0.6081	0.045*	
H10B	0.5159	0.7618	0.6203	0.045*	
C11	0.53530 (8)	0.83464 (12)	0.43762 (16)	0.0463 (4)	
H11A	0.5767	0.8387	0.3793	0.069*	
H11B	0.5221	0.9114	0.4656	0.069*	
H11C	0.4930	0.7995	0.3887	0.069*	

### Atomic displacement parameters (Å^2^)

**Table:**

	*U*^11^	*U*^22^	*U*^33^	*U*^12^	*U*^13^	*U*^23^
O1	0.0445 (5)	0.0230 (4)	0.0226 (4)	0.0049 (3)	−0.0062 (3)	0.0004 (3)
O2	0.0579 (6)	0.0364 (5)	0.0234 (4)	0.0062 (4)	−0.0031 (4)	−0.0042 (4)
O3	0.0402 (5)	0.0251 (4)	0.0273 (4)	0.0064 (3)	−0.0017 (3)	0.0001 (3)
N1	0.0353 (5)	0.0252 (5)	0.0214 (5)	−0.0008 (4)	−0.0014 (4)	0.0006 (4)
C1	0.0270 (5)	0.0233 (6)	0.0241 (5)	−0.0009 (4)	0.0031 (4)	0.0011 (4)
C2	0.0257 (5)	0.0250 (6)	0.0215 (5)	−0.0021 (4)	0.0025 (4)	0.0017 (4)
C3	0.0310 (6)	0.0254 (6)	0.0285 (6)	0.0003 (4)	0.0015 (4)	0.0041 (4)
C4	0.0381 (7)	0.0235 (6)	0.0327 (6)	0.0008 (5)	0.0018 (5)	−0.0033 (5)
C5	0.0359 (6)	0.0323 (6)	0.0242 (6)	0.0000 (5)	0.0010 (5)	−0.0049 (5)
C6	0.0320 (6)	0.0294 (6)	0.0217 (5)	0.0002 (4)	0.0000 (4)	0.0015 (4)
C7	0.0295 (6)	0.0229 (5)	0.0234 (5)	−0.0010 (4)	0.0020 (4)	0.0020 (4)
C8	0.0366 (6)	0.0258 (6)	0.0221 (5)	0.0030 (4)	−0.0036 (4)	0.0017 (4)
C9	0.0293 (6)	0.0280 (6)	0.0245 (5)	0.0003 (4)	−0.0018 (4)	−0.0001 (4)
C10	0.0436 (7)	0.0268 (6)	0.0432 (7)	0.0086 (5)	0.0032 (6)	−0.0040 (5)
C11	0.0472 (8)	0.0350 (7)	0.0578 (9)	0.0141 (6)	0.0135 (7)	0.0127 (7)

### Geometric parameters (Å, º)

**Table:**

O1—C1	1.3720 (14)	C4—H4	0.9500
O1—C8	1.4159 (13)	C5—C6	1.3867 (17)
O2—C9	1.1958 (14)	C5—H5	0.9500
O3—C9	1.3374 (14)	C6—H6	0.9500
O3—C10	1.4537 (14)	C7—H7	0.9500
N1—C7	1.2831 (14)	C8—C9	1.5070 (15)
N1—N1^i^	1.4121 (18)	C8—H8A	0.9900
C1—C6	1.3912 (15)	C8—H8B	0.9900
C1—C2	1.4046 (15)	C10—C11	1.5025 (19)
C2—C3	1.3991 (16)	C10—H10A	0.9900
C2—C7	1.4613 (15)	C10—H10B	0.9900
C3—C4	1.3827 (16)	C11—H11A	0.9800
C3—H3	0.9500	C11—H11B	0.9800
C4—C5	1.3901 (17)	C11—H11C	0.9800
			
C1—O1—C8	117.47 (9)	C2—C7—H7	118.9
C9—O3—C10	115.97 (9)	O1—C8—C9	107.59 (9)
C7—N1—N1^i^	111.26 (12)	O1—C8—H8A	110.2
O1—C1—C6	123.70 (10)	C9—C8—H8A	110.2
O1—C1—C2	115.44 (9)	O1—C8—H8B	110.2
C6—C1—C2	120.86 (10)	C9—C8—H8B	110.2
C3—C2—C1	118.49 (10)	H8A—C8—H8B	108.5
C3—C2—C7	122.39 (10)	O2—C9—O3	124.86 (11)
C1—C2—C7	119.11 (10)	O2—C9—C8	125.82 (11)
C4—C3—C2	121.01 (11)	O3—C9—C8	109.30 (9)
C4—C3—H3	119.5	O3—C10—C11	107.37 (11)
C2—C3—H3	119.5	O3—C10—H10A	110.2
C3—C4—C5	119.38 (11)	C11—C10—H10A	110.2
C3—C4—H4	120.3	O3—C10—H10B	110.2
C5—C4—H4	120.3	C11—C10—H10B	110.2
C6—C5—C4	121.16 (11)	H10A—C10—H10B	108.5
C6—C5—H5	119.4	C10—C11—H11A	109.5
C4—C5—H5	119.4	C10—C11—H11B	109.5
C5—C6—C1	119.07 (11)	H11A—C11—H11B	109.5
C5—C6—H6	120.5	C10—C11—H11C	109.5
C1—C6—H6	120.5	H11A—C11—H11C	109.5
N1—C7—C2	122.19 (10)	H11B—C11—H11C	109.5
N1—C7—H7	118.9		
			
C8—O1—C1—C6	5.25 (16)	O1—C1—C6—C5	−178.46 (10)
C8—O1—C1—C2	−174.91 (9)	C2—C1—C6—C5	1.71 (17)
O1—C1—C2—C3	178.99 (9)	N1^i^—N1—C7—C2	−179.14 (10)
C6—C1—C2—C3	−1.17 (16)	C3—C2—C7—N1	1.02 (17)
O1—C1—C2—C7	−1.15 (15)	C1—C2—C7—N1	−178.84 (10)
C6—C1—C2—C7	178.69 (10)	C1—O1—C8—C9	171.36 (9)
C1—C2—C3—C4	−0.39 (17)	C10—O3—C9—O2	3.46 (17)
C7—C2—C3—C4	179.75 (10)	C10—O3—C9—C8	−177.91 (10)
C2—C3—C4—C5	1.38 (18)	O1—C8—C9—O2	−11.11 (17)
C3—C4—C5—C6	−0.83 (18)	O1—C8—C9—O3	170.27 (9)
C4—C5—C6—C1	−0.70 (18)	C9—O3—C10—C11	176.28 (10)

Symmetry code: (i) −*x*+3/2, −*y*+1/2, −*z*+2.

### Hydrogen-bond geometry (Å, º)

**Table:**

*D*—H···*A*	*D*—H	H···*A*	*D*···*A*	*D*—H···*A*
C6—H6···O2^ii^	0.95	2.34	3.2802 (14)	168

Symmetry code: (ii) *x*, −*y*+1, *z*−1/2.
