# Supplementary figures and images for: Crystal structure of ethyl 2-[2-((1E)-{(1E)-2-[2-(2-eth­oxy-2-oxoeth­oxy)benzyl­idene]hydrazin-1-yl­idene}meth­yl)phen­oxy]acetate
Source: Acta Crystallogr E Crystallogr Commun. 2015 Jan 1;71(Pt 1):o16. doi: 10.1107/S2056989014025584 (PMC4331898; doi:10.1107/S2056989014025584)

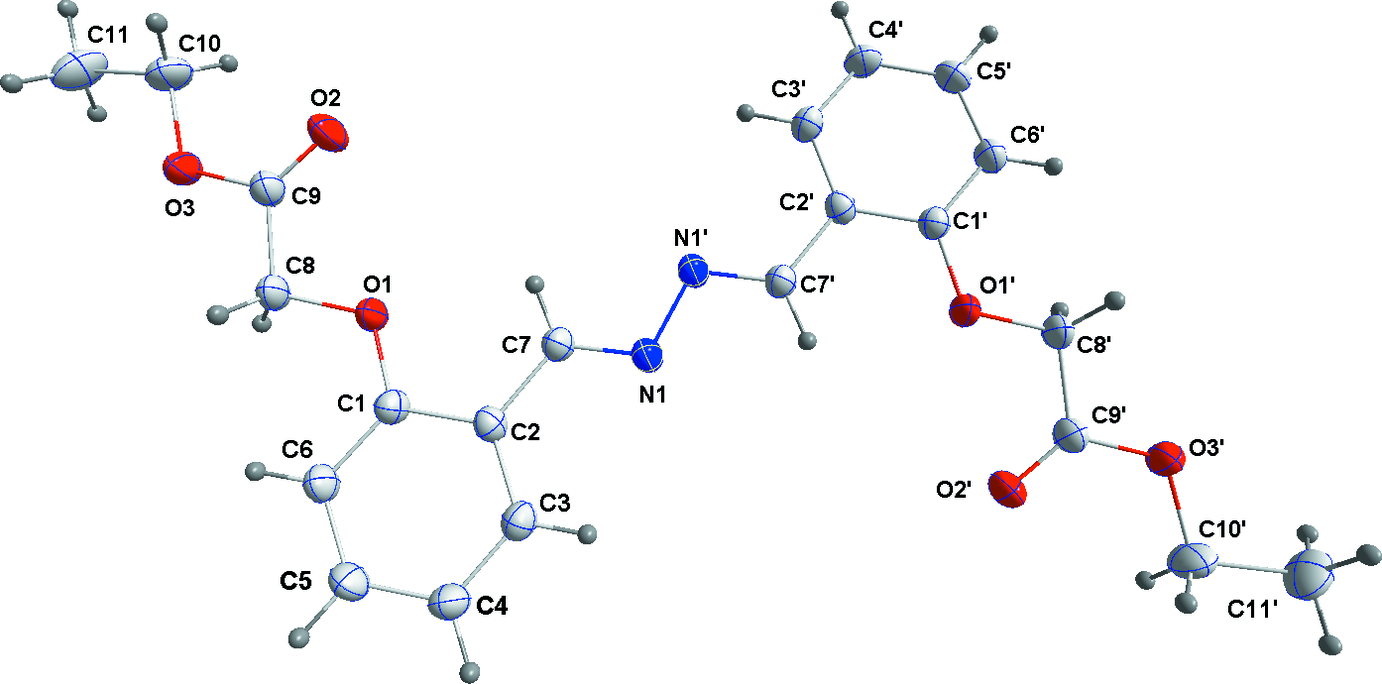

Supplement: Supplementary file 4 [file e-71-00o16-fig1.tif]

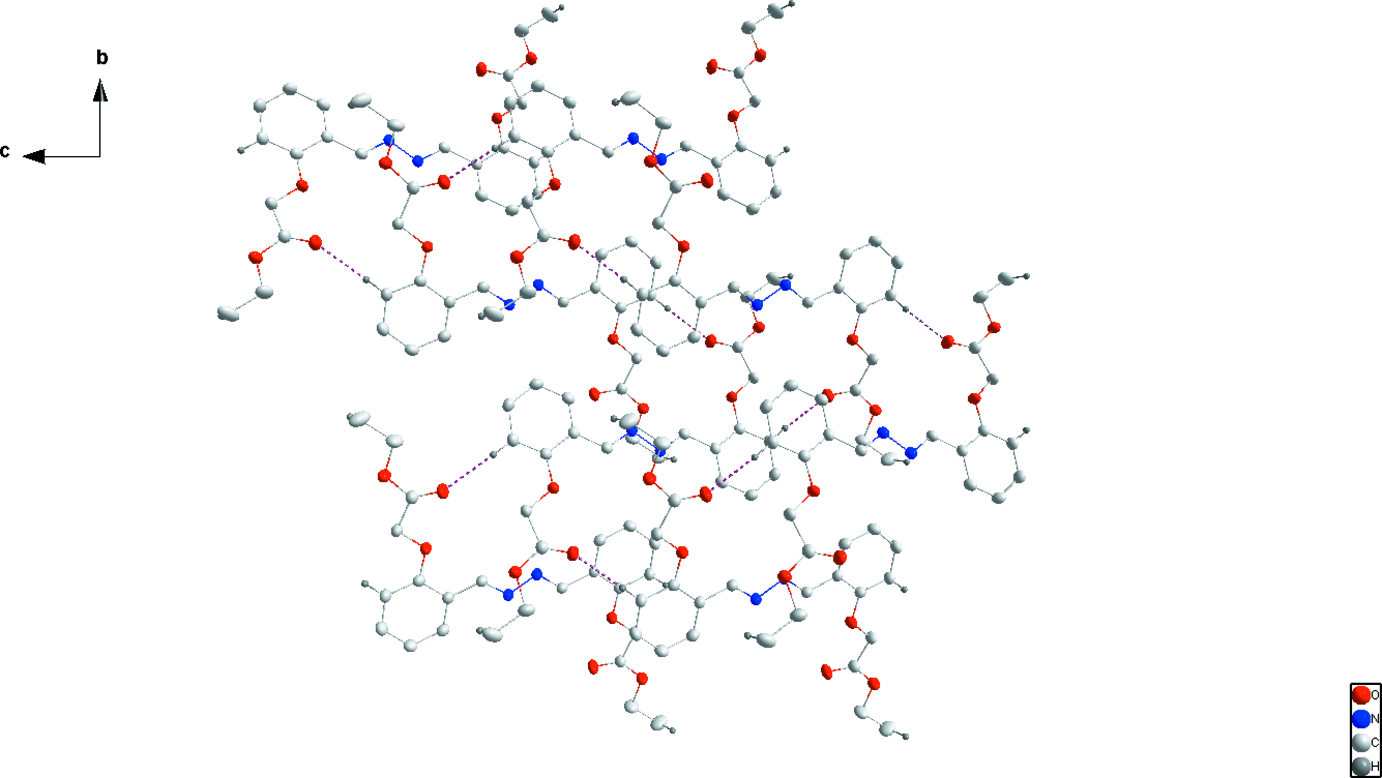

Supplement: Supplementary file 5 [file e-71-00o16-fig2.tif]

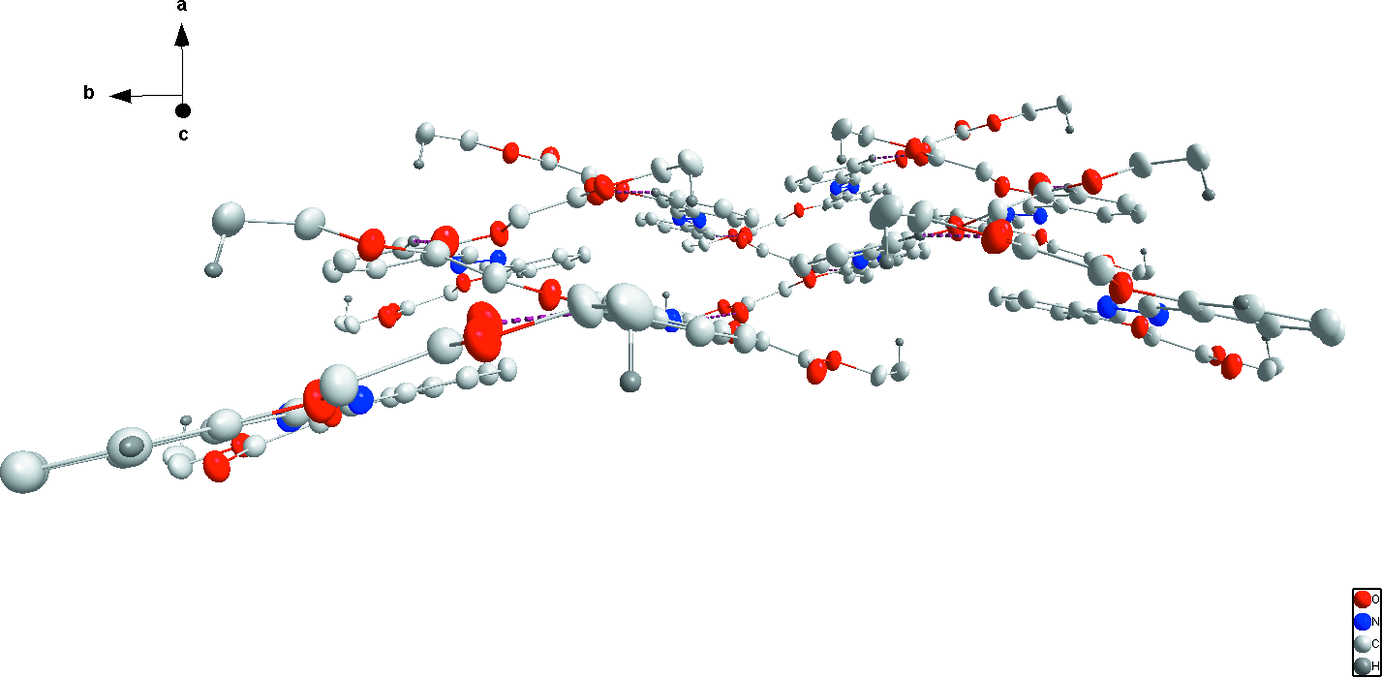

Supplement: Supplementary file 6 [file e-71-00o16-fig3.tif]
